# The household economic costs associated with depression symptoms: A cross-sectional household study conducted in the North West province of South Africa

**DOI:** 10.1371/journal.pone.0224799

**Published:** 2019-11-05

**Authors:** Sumaiyah Docrat, Susan Cleary, Dan Chisholm, Crick Lund

**Affiliations:** 1 Alan J. Flisher Centre for Public Mental Health, Department of Psychiatry and Mental Health, University of Cape Town, Cape Town, South Africa; 2 Health Economics Unit, University of Cape Town, Cape Town, South Africa; 3 Department of Mental Health and Substance Abuse, World Health Organization, Geneva, Switzerland; 4 Centre for Global Mental Health, King’s Global Health Institute, Health Service and Population Research Department, Institute of Psychiatry, Psychology and Neuroscience, King’s College London, London, England, United Kingdom; Medical Research Council, SOUTH AFRICA

## Abstract

**Aim:**

The aim of this study was to assess the association between depression symptom severity and household income, consumption, asset-based wealth, debt and use of distress financing strategies, to understand how depression symptom severity and household economic welfare are related.

**Methods:**

A household survey was administered to the households of primary health clinic-attenders who were screened for depression symptoms using the 9-item Patient Health Questionnaire in the chronic care units of four primary health clinics in the North West province of South Africa. Univariate and multivariable regression models were used to assess whether a range of household economic measures were significant predictors of depression symptom severity; and whether depression symptom severity significantly predicted changes to household economic welfare, across a number of different economic measures using both multiple linear regression and logistic regression analyses.

**Results:**

On univariate analysis, certain characteristics were associated with significantly worse (higher) PHQ-9 scores, namely: households in which the household head was younger, female, and unmarried; households in which the indexed patient was younger, and did not receive an education beyond primary school; increasing household size, receipt of a social grant, households living in housing constructed of metal sheet walls and households making use of a public tap as their primary water source. In addition, univariate analysis demonstrated that higher log-transformed food expenditure, lower log-transformed capacity to pay, the presence of household debt and both reducing the size or frequency of meals and drawing up retail shop accounts in response to financial distress over the past three years were associated with significantly worse (higher) PHQ-9 scores. Multivariable analysis demonstrated that larger household sizes (p<0.05), receipt of social grants (p<0.05), higher food expenditure (p<0.01), and drawing up retail shop accounts in response to financial distress (p<0.05) were independently predictive of worse (higher) PHQ-9 scores. Inversely, increasing age of the household head (p<0.05), having piped water directly into the household (as opposed to making use of a public water sources) (p<0.01), and increasing capacity to pay (p<0.01) were independently predictive of better (lower) PHQ-9 scores. Similarly, multivariable analysis demonstrated that worse (higher) PHQ-9 scores were independently predictive of lower household capacity to pay (p<0.10) and higher food expenditure (p<0.01).

**Conclusions:**

This study is the first of its kind in South Africa, identifying household economic factors associated with increased depression symptom severity on a continuum; and demonstrating that financial risk protection efforts are needed across this continuum. The study demonstrates that the relationship between poverty and mental health extends beyond the individual to affect household economic functioning. These findings must be included in policy considerations to achieve effective protection for vulnerable households facing the interaction of depression and adverse economic circumstances.

## Introduction

Depression is a leading cause of disability worldwide [1–4]. In part, this is explained by the high treatment gap globally; 12-month prevalence rates for major depression and anxiety disorders stood at 4.6% and 9.8% of the global population in 2017, with the treatment gap for minimally adequate treatment exceeding 80% (83.5% and 90.2%, for major depression and anxiety respectively) [5, 6]. Since 2010, South Africa’s prevalence of major depression and anxiety disorders has exceeded global averages; in 2017, the prevalence stood at 6.7%, representing one of the top five contributors to years lived with disability (YLD) in the country [7]. As in South Africa, other low- and middle-income countries (LMICs) experiencing demographic and epidemiological transitions are realizing the increasing public health importance of common mental disorders, including depression [3, 8].

A strong association exists between depression and poverty [9]. Two causal pathways are hypothesized to maintain the cycle of poverty and mental illness: the *social causation* hypothesis, by which the conditions associated with poverty (such as increased stress, poor housing, social exclusion, reduced social capital, malnutrition and increased violence and trauma) increase the risk for mental illness; and the *social selection* or *social drift* hypothesis, by which people living with mental illness are at increased risk of drifting into or remaining in poverty as a result of increased healthcare expenditure, reduced productivity, stigma and job loss [9, 10]. Until recently, the limited availability of longitudinal data means that little was known regarding the causal relationships underlying these associations. Earlier studies suggested that there was more promising evidence that the social causation hypothesis was more applicable to depression (i.e. that conditions associated with poverty increase the risk of depression), however a recent study on poverty and depression, conducted using three waves of a nationally representative longitudinal dataset in South Africa, demonstrated that both social causation and social drift act simultaneously [3]. Household-level data which examines the economic impact of depression on households is limited for LMICs; in a 2010 systematic review of poverty and common mental disorders in LMIC, the vast majority of the 115 studies examined individual-level rather than household-level economic variables [9].

Among LMICs, the costs of illness do not fall on ill individuals alone; the time and financial costs of illness are often carried by healthy household members and decisions about treatment seeking and coping with financial difficulty are similarly made at the household-level [11–13]. The economic impact of physical illnesses on households in LMICs has been well documented [11, 14, 15]. Unanticipated increases in health expenditures coupled with a reduction of functional capacity and lost income as a result of reduced productivity from illness, or death of the main household income earner, is considered a primary risk factor for impoverishment—a phenomenon known as the *medical poverty trap* [14–19]. Households risk worsening health by adapting their use of healthcare and other subsistence needs to evade costs they cannot face, or by employing financial strategies which compromise their livelihoods [12, 16, 17, 20]. The need for evidence quantifying the magnitude of the economic impact of illness to individuals and households is crucial in the context of the recent adoption of the Sustainable Development Goals (SDGs), specifically the inclusion of universal health coverage (UHC) goals which include a commitment by governments to protect vulnerable households against the catastrophic financial and economic consequences of illness [21].

Depression is characterized by a wide range of emotional, cognitive, physical and behavioral symptoms. Over several decades there has been debate about whether sub-threshold depression symptoms (i.e. below the threshold for a clinical diagnosis of depression) are associated with significant psychosocial impairment, and relatedly, whether sub-threshold depression symptoms share the same constructs with diagnosable major depression [22, 23]. However, in recent years, there is increased recognition that the symptoms of depression must be considered on a continuum, and clinical depression should not be considered categorically distinct from other degrees of depression symptoms [22, 23]. Further, the common symptoms of mental distress such as anxiety or low mood have been associated with more total disability at a population-level than diagnostically defined mental disorders [24].

A recent Lancet Commission on Global Mental Health and Sustainable Development emphasized the need to adopt a dimensional approach to the classification and treatment of mental disorders by moving beyond absolute boundaries which denote the presence or absence of a mental disorder [24]. The Commission asserts that the lengthy period between the appearance of initial symptoms, characterized by a gradual decline in functioning, is often the time when early interventions can lead to better outcomes (as opposed to waiting until the disease has progressed and symptoms have persisted sufficiently to warrant a diagnosis). With this in mind, a broader research agenda is required to address key questions around the appropriate treatment and prevention of depression, which acknowledges the importance of management of sub-threshold symptoms to mitigate progression to more serious depression and, of relevance to this paper, their potential broader economic impacts. This study therefore aims to assess the association between depression symptom severity and household income, consumption, asset-based wealth, debt and use of distress financing strategies, and to understand how depression symptom severity and household economic welfare are related, based on insights from a survey conducted in a South African setting.

## Materials and methods

### Study design

This study forms part of the Emerald (Emerging mental health systems in low- and middle-income countries) project which pursued a programme of research into a number of mental health system strengthening components across six LMICs (Ethiopia, India, Nepal, Nigeria, South Africa and Uganda), [16]. As part of the mental health financing component of the project, a household survey was carried out in each of the six Emerald country sites to determine the economic consequences of mental disorders to households. In South Africa, the cross-sectional household survey was conducted in the Dr. Kenneth Kaunda (Dr. KK) health district of the North West province. This study adheres to the STROBE guidelines for the reporting of observational cross-sectional epidemiological studies [25].

### Setting

The rationale for the choice of the Dr. KK health district (North West province), as well as the district characteristics, has been described in detail elsewhere [26–29]. Briefly, the Dr. KK district was identified based on the priorities identified by the Department of Health (DOH). The district is also serving as a pilot site for the implementation of a new mental health care plan, being conducted through a separate, ethically approved study: the PRogramme for Improving Mental health carE (PRIME) [30], to which the Emerald household survey recruitment was linked. Dr. KK comprises a population of 745,878, with an unemployment rate of 30.4%; above the provincial and national averages and is estimated to be 14% rural [27, 31]. Dr. KK faces a high prevalence of both HIV (30% of the district population) and Tuberculosis (TB), and a rising burden of concomitant non-communicable disease including diabetes and hypertension [27]. Although the district has one specialized psychiatric hospital, four general hospitals with capacity for acute admissions for severe psychiatric cases and a multi-disciplinary team providing outpatient care for people with severe mental disorders; a situational analysis conducted in 2014 revealed that the district is unable to meet the mental health needs of the district population [27].

### Data collection and sample

Between August 2014 and July 2015, individual-level screening of adult (≥ 18 years) primary health care (PHC) attenders in the chronic care units of four PHC clinics was conducted through the PRIME Cohort Study [32]. PHC attenders were screened by PRIME researchers following their consultation with a clinician using the PHQ-9 [33], which has been widely used in LMICs and validated in primary care patients in South Africa [32, 34]. Psychometric assessment of the tool has indicated it has good validity and reliability [33, 35]. PHQ-9 scores ranging from 0–4, 5–9, 10–14, 15–19 and 20–27 are considered to indicate minimal, mild, moderate, moderately-severe and severe depressive symptoms, respectively [32, 34]. A threshold score of 10 identifies a probable case of major depression [32, 34]. After screening, participants were approached and permission to visit their households for the Emerald study was sought through written informed consent, irrespective of their PHQ-9 scores.

Individuals who provided written informed consent were visited in their households by Emerald fieldworkers where the head of the household, or adult most knowledgeable about the household financial situation, was asked to participate in the household study by providing additional written informed consent. A household was defined as individuals living in the same home, who shared a common source of food. Where the household informant consented, fieldworkers administered a household survey, lasting approximately one hour, in English or Setswana, the languages of the majority in Dr. KK. The survey instrument is adapted from the previously validated World Health Organization (WHO) Study on global AGEing and adult health (SAGE) survey on health and ageing developed specifically for use in LMICs [36]. SAGE has adapted and added to the methods and instruments developed by the WHO for the World Health Survey (WHS) that was conducted in 2002 and 2003 in 70 countries [37]. The key domains of the SAGE household instrument are as follows: demographics of household members, housing (type and ownership of housing, number of residents); transfers (to or from those not living in household, including financial or non-financial help to and from family and friends, as well as state benefits, debts or loans); assets and income (asset index, sources and levels of income); expenditure (food and non-food items, health care costs and source of funds for these expenditures); and the global situation (financial strain index, perceived situation) [28, 36]. Demographic data related to the indexed PHC attender were obtained from the PRIME Cohort study [32].

The broader Emerald household study sought to describe the economic characteristics of households affected by depression symptoms that had met the threshold for major depression; compared to those that did not meet the threshold [28]. Previous cross-country analyses of these data therefore did not consider depression symptoms on a continuum nor did they include any in-depth regression analyses to determine which factors were associated with worse economic circumstances for households affected by depression [28]. Further, previous cross-country analyses of these data included households in which indexed PHC attenders were diagnosed with depression but screened-negative using the PHQ-9 (i.e. disagreement between PHC-worker diagnoses and PRIME researcher screening); households in which indexed PHC attenders were screened during the PRIME pilot recruitment period (whereby evidence emerged of fieldworker error in the administration of the PHQ-9), and; households in which indexed PHC attenders were screened at subsequent PHC-visits during the recruitment period and there was disagreement in their screening scores [32].

### Measures

The primary economic outcome measures were: household income, consumption, capacity to pay, food expenditure, the presence of household debt, household asset score and household use of distress financing strategies in response to financial difficulty. A detailed description of the construction and assumptions used for each of these economic measures is provided in S1 File and elsewhere [28]. Briefly, household reports of income by source and consumption by item were standardized to reflect annual amounts during the data cleaning process, given that a range of recall periods were applied depending on the income source or consumption item [28].

Total household food (subsistence) consumption was subtracted from total household consumption as a measure of *households’ capacity to pay*. These financial variables were adjusted for household size and composition to ensure all comparisons generated would be based on a per adult equivalency (per capita) basis, using the OECD modified scale, accounting for the varying resource needs of adults and children in the household, and the economies of scale associated with sharing household resources [28, 38]. All financial data were converted to United States Dollars (USD) using the 2015 average annual exchange rate (the year data collection was conducted) reported by the US Department of Treasury for South Africa (1 USD = ZAR 13.46) [39]

A range of household assets were used to generate a *household asset score* using Multiple Correspondence Analysis (MCA). MCA as opposed to Principal Components Analysis (PCA) was used to create the asset index as MCA makes fewer assumptions about the underlying distributions of indicator variables and is more suited for the analysis of categorical variables [28, 40–42]. Wealth quintiles were generated based on these scores for descriptive purposes. For the assessment of *household-use of distress financing strategies in response to financial difficulty*, summary variables were generated based on the household report of: withdrawing children from school, reducing healthcare use, restricting the size or frequency of meals, or drawing up accounts at retail outlets in response to financial distress over the past three years. Similarly, for the assessment of the presence of *household debt*, summary variables were generated based on the report of debt in the household.

### Data analysis

We used frequency distributions and univariate descriptive statistics for preliminary analysis, to describe household head, indexed patient, and household and housing characteristics among the sampled households. Mean PHQ-9 scores and standard deviations (SD) were reported for each characteristic. To assess independent differences in depressive symptom severity (PHQ-9 scores) across these characteristics, *p-*values were calculated using: two-sample, unpaired t-tests for dichotomous categorical variables; one-way analyses of variance (ANOVA) for categorical variables with more than 2 groups, and; linear regression for continuous variables. Tests were considered significant if *p*-values were less than 0.05 (5% level). Where depressive symptom severity (PHQ-9 score) was significantly associated with socio-demographic factors, these were adjusted for in all multivariable regression models.

Univariate and multivariable models were used to assess risk factors predictive of worse (higher) PHQ-9 scores. Independent variables included significant socio-demographic factors, household income, consumption, capacity to pay, food expenditure, the presence of household debt, asset-based wealth score and use of distress financing strategies in response to financial difficulty. Predictor variables that retained a value of p<0.05 on univariate analysis were entered into the multivariable linear regression model. As anticipated, financial data reported by households were highly skewed to the right. For all regressions, these data were logarithm transformed to fulfill the assumption of normality required for the use of parametric tests. The assumptions justifying the use of linear regression were evaluated; whilst no collinearity was found between the predictor variables, robust-standard errors were included in the regression model to account for the non-homogenous variance of the residuals.

Similarly, a series of multiple linear regression models were fitted to assess whether depression symptoms independently predicted lower household income, consumption, capacity to pay, food expenditure and asset-based wealth scores derived through MCA. Each model proceeded by initially assessing the effect of PHQ-9 score independently through linear regression, with log-transformed financial variables and asset-based wealth scores treated as the continuous response variables. Where PHQ-9 scores retained a value of p<0.05 on univariate analysis for each economic outcome, multivariable linear regression models were fit, adjusting for significant socio-demographic factors.

Finally, logistic regression analyses were used to assess the independent effect of depression symptom severity on the presence of debt in the household (coded dichotomously as yes or no) and of the use of distress financing strategies in response to financial difficulty (each strategy coded dichotomously as yes or no). All models were adjusted for independent baseline associates.

To account for sampling errors in the estimated variance of covariates included in the final models, bootstrapping was applied. Bootstrapping was run on the models with 1000 replacements to obtain the final estimates that are reported. For all multiple linear regression models, the final adjusted regression coefficients and 95% confidence intervals are reported; for all logistic regression models, the final odds ratios and 95% confidence intervals are reported.

To avoid the loss of statistical power in detecting the relationship between depression symptom severity and the economic variables of interest, households were *only* categorized based on depression symptom severity cutoffs (i.e. minor, mild, moderate, moderate-severe and severe depressive symptoms) for the purpose of presenting median household income, consumption, capacity to pay, and; overall frequencies in the use of distress financing strategies, asset-based wealth group assignment and the presence of debt in the household. Medians (as opposed to means) were reported in the case of financial variables given that these data were skewed and non-parametric. In all univariate and multivariable models, depression symptom severity (PHQ-9 score) was included as a continuous variable.

### Ethical considerations

The consenting process for the study involved has already been described. All participants provided voluntary informed consent to participate in the study, none of the household heads or individual household members screened at the PHC facility lacked capacity to consent. Hand-held electronic data-collection devices were used to collect household data which ensured that data remained secure by (a) password-protecting access to the hand-held devices, (b) transmitting data to the server regularly, (c) password-protecting the computers that accessed the server, and (d) accessing raw data on the server via a password-protected website. The study including all consent procedures received ethical approval from the ethics review committees of the University of Cape Town (HREC REF 531/2013), as well as that of the project coordinating centre (King’s College London) and WHO (RPC619).

## Results

### Sample characteristics

**Fig 1:**
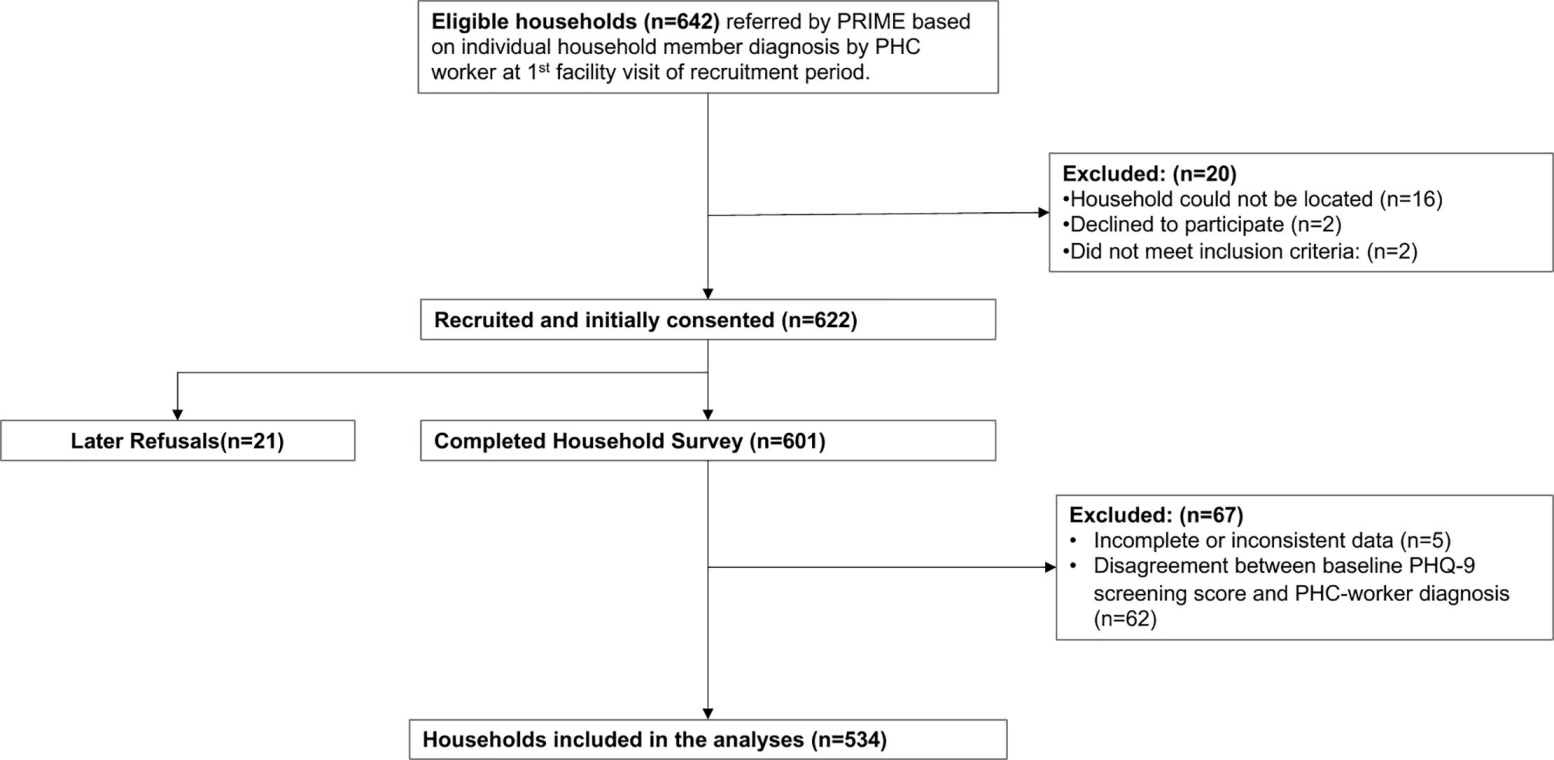
Participant flow diagram.

A total of 534 households were included in the analyses (Fig 1). The final sample size was derived after the removal of households who had participated in the survey but had >80% incomplete data (i.e. only the household roster was completed) (n = 5), and; the removal of households in which there was disagreement between baseline PHQ-9 score and PHC-worker diagnosis (n = 62). Prior to exclusion, excluded households were assessed to ensure that they did not have any significant differences with respect to their socio-demographic characteristics, when compared to households that were included in the final analyses (S1 Table).

Across the sampled households, 46% (n = 248) included a household member who achieved a PHQ-9 score of 10 or more, indicating probable cases of major depression; 47.7% (n = 255) included a household member who had minimal or mild depressive symptoms, whilst for the remaining 5.8% (n = 31) of households, the indexed household member had no depressive symptoms (Table 1). Female-headed households represented 52.1% of the sample, with the majority of household heads being unmarried (73%) with no formal education beyond primary school (80.0%). Indexed patients within households were predominantly female (78.5%), with children (86.6%) and similarly unmarried (79.1%) with a primary school education or less (87.4%). The mean household size consisted of four household members, with 73% of households receiving a social grant, although the detail of the specific type of grant received was not requested from participants. It is important to note that South Africa provides a range of different grants including the Child Support Grant, Older Person’s Grant, Disability Grant, Grant-in-Aid, Care Dependency Grant, War Veteran’s Grant, Foster Child Grant [43]. Only 1.7% of the sampled households included a household member with health insurance. With respect to housing, the majority of households were residing in housing that was provided free of charge (i.e. government housing) (61.1%), with 32.2% residing in housing that was owned by the household head. The majority of the sample lived in housing with cement walls, with 11.4% of households living in structures constructed of metal sheet walls. Just over half of the households had water piped directly into their dwelling (50.9%) with the remaining households accessing water through public taps or piped water into a yard.

**Table 1:** Sociodemographic characteristics and depression symptom (PHQ-9) scores among the sampled households.

Characteristics	N or Mean	% or SD	PHQ-9 Score		Comparisons (p-values)[Table-fn t001fn001]
			Mean	SD	
**Household Head characteristics**					
Age					**0.053**
20–35	60	11.2	9.6	5.9	
36–50	197	36.9	9.1	5.8	
51–65	206	38.6	8.2	5.3	
66–80	63	11.8	7.3	5.0	
>81	8	1.5	10.6	4.0	
Sex					**0.009**
Male	256	47.9	7.8	5.5	
Female	278	52.1	9.1	5.5	
Marital Status					**0.006**
Unmarried	390	73.0	8.9	5.4	
Married	144	27.0	7.3	5.6	
Education					0.239
Primary school or less	427	80.0	8.6	5.5	
Beyond primary school	107	20.0	8.0	5.6	
**Indexed Patient characteristics**					
Age					**0.042**
20–35	118	22.1	9.1	5.2	
36–50	192	36.0	9.0	5.9	
51–65	177	33.1	8.3	5.5	
66–80	41	7.7	7.3	4.6	
>81	6	1.1	6.0	1.4	
Sex					0.181
Male	114	21.5	8.0	5.8	
Female	416	78.5	8.6	5.4	
Marital Status					0.061
Unmarried	419	79.1	8.7	5.5	
Married	111	20.9	7.6	5.5	
Education					**0.040**
Primary school or less	463	87.4	8.6	5.5	
Beyond primary school	67	12.6	7.4	5.3	
Children					0.765
No children	71	13.4	8.6	5.2	
Has children	457	86.6	8.4	5.5	
Depressive symptom severity					**0.028**
None	31	5.8%	0.0	0.0	
Minimal	116	21.7%	2.7	1.1	
Mild	139	26.0%	6.4	1.2	
Moderate	166	31.1%	11.5	1.4	
Moderately-severe	64	12.0%	16.7	1.3	
Severe	18	3.4%	21.4	1.5	
**Household characteristics**					
Household Size					**0.001**
1–2	134	25.1	7.7	5.3	
3–4	213	39.9	8.5	5.3	
5–6	131	24.5	8.8	5.9	
7–8	44	8.2	11.1	5.7	
>8	12	2.2	10.3	4.6	
Health Insurance Coverage					0.258
Uninsured	525	98.3	8.5	5.5	
Insured	9	1.7	6.3	4.6	
Social Protection					**0.001**
Not receiving social grant	144	27.0	7.2	5.2	
Receiving social grant	390	73.0	8.9	5.5	
**Housing characteristics**					
Ownership					0.199
Owned and fully paid off	165	32.2	8.5	5.1	
Provided free of charge	313	61.1	8.7	5.5	
Rented	34	6.7	6.9	6.9	
Wall Material					**0.050**
Metal sheet	61	11.4	9.6	5.4	
Cement	473	88.6	8.3	5.5	
Water Source					**0.000**
Piped into dwelling	272	50.9	7.4	5.0	
Piped into Yard or Public Tap	262	49.1	9.6	5.8	

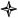
two-sample, unpaired t-test for dichotomous categorical variables, one-way analysis of variance (ANOVA) for categorical variables with > 2 groups, linear regression for continuous variables

### Median absolute income, consumption and capacity to pay by severity-group

**Fig 2:**
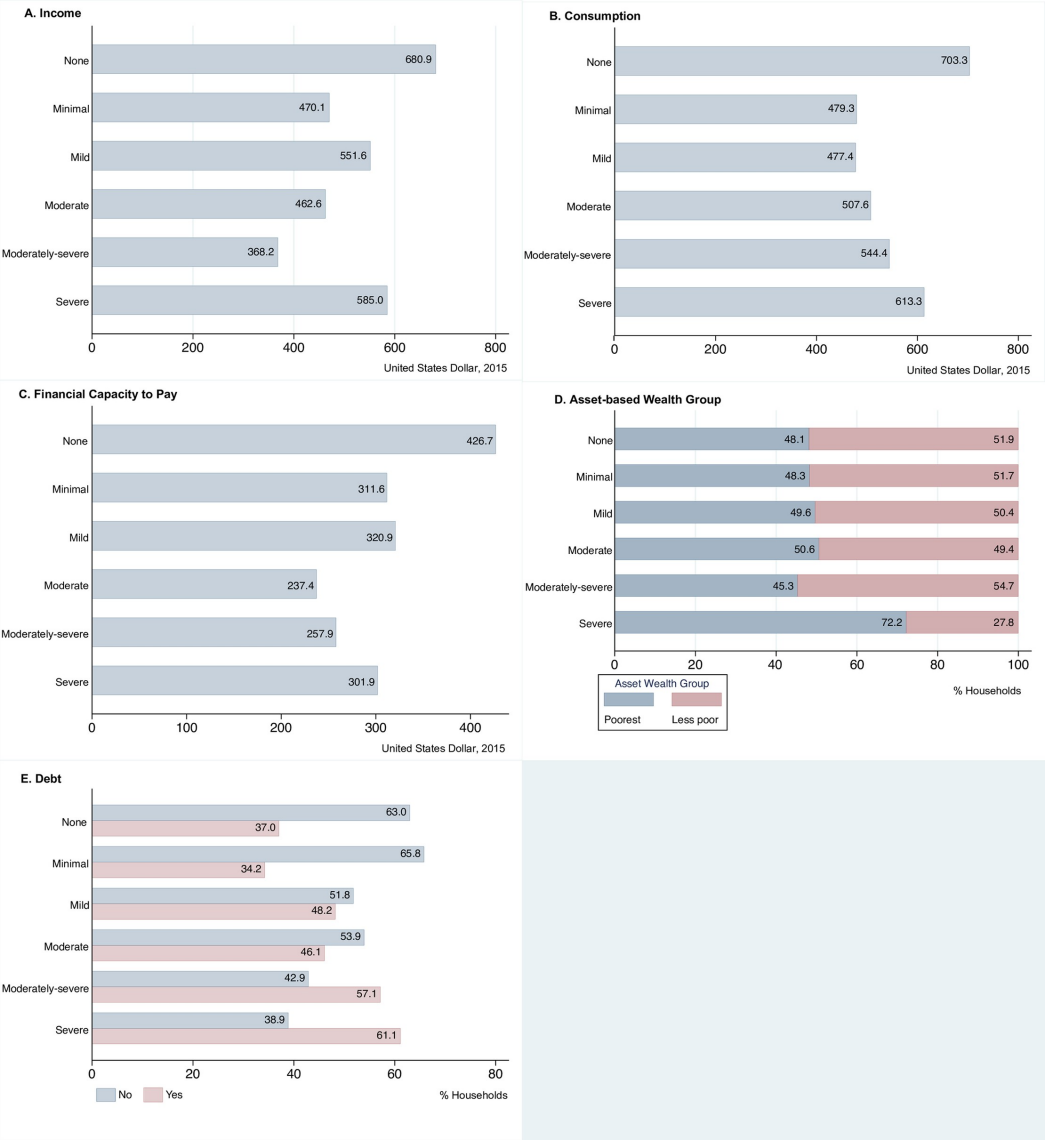
Annual median household (A) income, (B) consumption and (C) capacity to pay (per adult equivalent) and frequency distributions of (D) asset-based wealth and (E) debt among the sampled households, by depression symptom severity group.

In absolute terms, median annual household income ranged from USD680.9 per adult equivalent amongst households unaffected by depression symptoms to USD368.2 per adult equivalent amongst households affected by moderately-severe depression symptoms (Fig 2A). Whilst there was not a consistent trend of decreasing income by increasing depression symptom severity group, when compared with those unaffected by depressive symptoms, households affected by minimal, mild, moderate, moderately-severe and severe depressive symptoms all reported lower median household incomes in absolute terms.

With regards to household consumption, households unaffected by depression symptoms reported median annual household consumption per adult equivalent of USD703.3 (Fig 2B). Annual consumption per adult equivalent ranged from USD479.3 amongst households affected by minimal depression symptoms to USD613.3 amongst households affected by severe depression symptoms. Although households affected by any form of depression symptoms all reported lower median household consumption when compared to households unaffected by depression symptoms; median household consumption (in absolute terms) increased with depression symptom severity grouping.

Median annual capacity to pay ranged from USD426.7 per adult equivalent amongst households unaffected by depression symptoms to USD237.4 per adult equivalent amongst households affected by moderate depression symptoms (Fig 2C). While there was no linear relationship between capacity to pay and increasing depression symptom severity group, capacity to pay appears to decrease as depression severity increases; amongst all depression symptom severity groups, median annual capacity to pay was lower than those unaffected by depression symptoms.

### Asset-based wealth

With regards to asset-based wealth, a higher proportion of households were assigned to the poorest wealth group amongst households affected by severe depression symptoms (72.2%), compared with those unaffected by depression symptoms where 48.1% of households were assigned to poorest wealth group (Fig 2D). This trend was consistent amongst the minimal, mild and moderate depression symptom severity groups; as depression symptom severity increased, a larger proportion of households were assigned to the poorest wealth group. The only exception to this trend was amongst households affected by moderately-severe depression symptoms, where 54.7% of households were assigned to the less poor wealth group and consequently, 45.3% assigned to the poorest wealth group.

### Debt affecting households

With regards to the presence of debt in the household, the proportion of households reporting that they have current debts ranged from 34.2% of households affected by minimal depression symptoms, to 61.1% of households affected by severe depression symptoms (Fig 2E). Particularly for households affected by moderate, moderately-severe and severe depression symptoms (i.e. PHQ-9 scores≥10), the proportion of households reporting debt increased with depression symptom severity.

### Coping with financial distress

Across the sampled households, 17.8% (n = 95) reported reducing the frequency or size of meals in response to financial difficulty over the past three years (Table 2). There were very small numbers of households reducing their use of healthcare (n = 2) or withdrawing children from school (n = 5) in response to financial difficulty. Nonetheless, 11% (n = 59) of the sampled households reported that they had drawn up accounts at retail shop outlets in response to financial difficulty over the past three years.

**Table 2:** Use of distress financing strategies and depression symptom (PHQ-9) scores among the sampled households.

Use of Distress Financing Strategies in response to financial difficulty	N	%	Depressive Symptom (PHQ-9) Score		Comparisons (p-values)[Table-fn t002fn001]
			Mean	SD	
**Reduce frequency or size of meals**					**0.007**
No	439	82.2	8.3	5.5	
Yes	95	17.8	10	5.4	
**Reduce use of healthcare**					0.774
No	532	99.6	8.6	5.5	
Yes	2	0.4	7.5	4.9	
**Withdraw children from school**					0.335
No	529	99.1	8.6	5.5	
Yes	5	0.9	11	4.3	
**Draw up retail shop accounts**					**0.025**
No	475	89	8.4	5.4	
Yes	59	11.0	10.2	6.4	

^
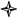
^two-sample, unpaired t-test for dichotomous categorical variables

### Univariate analyses

On univariate analysis, certain characteristics were associated with worse (higher) PHQ-9 scores, namely: age (p = 0.011), gender (p = 0.009) and marital status (p = 0.006) of the household head; age (p = 0.029) and education-level (p = 0.040) of the indexed patient; the household size (p = 0.000), whether the household received a social grant (p = 0.001), the wall material of the housing (p = 0.050), household water source (p = 0.000), higher log-transformed food expenditure (p = 0.006), lower log-transformed capacity to pay (p = 0.035), the presence of household debt (p = 0.006) and both reducing the size or frequency of meals (p = 0.007) and drawing up retail shop accounts (p = 0.025) in response to financial distress over the past three years. Univariate analyses also found that higher PHQ-9 scores were associated with the presence of household debt (p = 0.006) and both reducing the size or frequency of meals (p = 0.007) and drawing up retail shop accounts (p = 0.025) in response to financial distress over the past three years.

### Multivariable analyses

In model 1, which had depressive symptoms as the dependent variable, multivariable analysis demonstrated that larger household sizes (p<0.05), receipt of social grants (p<0.05), higher food expenditure (p<0.01), and drawing up retail shop accounts in response to financial distress (p<0.05) were independently associated with worse (higher) PHQ-9 scores (Table 3). Inversely, increasing age of the household head (p<0.05), having piped water directly into the household (as opposed to making use of a public water sources) (p<0.01), and increasing capacity to pay (p<0.01) were independently associated with better (lower) PHQ-9 scores. For a one unit increase in household head age, PHQ-9 scores decreased by 0.043; whilst a one unit increase in household size increased PHQ-9 scores by 0.323. In comparison to households not receiving social grants, PHQ-9 scores of households receiving grants were 1.19 times higher. Interestingly, compared to households making use of public water sources, households with water piped directly into the household had PHQ-9 scores that were 1.93 times lower. With regards to the financial predictors of interest, for a 10% increase in household’s capacity to pay, expected mean PHQ-9 scores decrease by 0.06 whilst for a 10% increase in household food expenditure, expected mean PHQ-9 scores increase by 0.08. In comparison to households that did not draw up retail shop accounts in response to financial distress, households that did had PHQ-9 scores that were 1.75 times higher. While indexed patient age and education was not significantly associated with depression scores at the 95% interval, there appears to be a protective association with both increased education and age on depression scores (i.e. at the 90% confidence level).

**Table 3:** Multivariable linear and logistic regression models.

	Model 1^a^	Model 2^a^	Model 3^a^	Model 4^b^	Model 5^b^	Model 6^b^
**Multivariable predictor**						
**Household head age**^**c**^						
*Adjusted Coefficient or Odds Ratio (95% CI)*	-0.043 (-0.083,-0.004)	0.002 (-0.004,0.007)	-0.001 (-0.009,0.006)	0.999 (0.982,1.017)	0.977 (0.958,0.996)	0.989 (0.958,1.022)
*p-value*	0.032*	0.563	0.705	0.946	0.019*	0.521
**Household head sex** *(female vs male)*						
*Adjusted Coefficient or Odds Ratio (95% CI)*	0.552 (-0.551,1.654)	-0.044 (-0.203,0.115)	-0.141 (-0.339,0.057)	0.870 (0.534,1.419)	0.987 (0.548,1.776)	1.482 (0.588,3.735)
*p-value*	0.327	0.586	0.162	0.577	0.964	0.404
**Household head marital status** *(married vs unmarried)*						
*Adjusted Coefficient or Odds Ratio (95% CI)*	-0.738 (-2.055,0.580)	0.258 (0.071,0.445)	0.015 (-0.228,0.259)	1.010 (0.558,1.830)	0.503 (0.233,1.086)	1.372 (0.481,3.911)
*p-value*	0.273	0.007**	0.901	0.973	0.080	0.555
**Indexed patient age**^**c**^						
*Adjusted Coefficient or Odds Ratio (95% CI)*	-0.029 (-0.063,0.005)	-0.001 (-0.006,0.004)	0.001 (-0.006,0.008)	1.004 (0.988,1.021)	0.998 (0.976,1.021)	1.003 (0.974,1.033)
*p-value*	0.093	0.630	0.780	0.612	0.866	0.846
**Indexed patient education** *(beyond primary school vs primary school or less)*						
*Adjusted Coefficient or Odds Ratio (95% CI)*	-1.307 (-2.785,0.171)	0.074 (-0.145,0.293)	-0.043 (-0.317,0.231)	1.132 (0.592,2.167)	0.998 (0.976,1.021)	0.586 (0.174,1.974)
*p-value*	0.083	0.507	0.758	0.707	0.083	0.389
**Household size**^**c**^						
*Adjusted Coefficient or Odds Ratio (95% CI)*	0.323 (0.075,0.572)	-0.063 (-0.101,-0.026)	-0.045 (-0.093,0.003)	1.136 (1.018,1.267)	1.094 (0.965,1.239)	1.094 (0.918,1.304)
*p-value*	0.011*	0.001**	0.067	0.023*	0.161	0.315
**Social Protection** *(receiving grant vs*. *not receiving grant)*						
*Adjusted Coefficient or Odds Ratio (95% CI)*	1.190 (0.076,2.304)	-0.069 (-0.256,0.119)	-0.054 (-0.266,0.157)	1.439 (0.817,2.533)	1.268 (0.629,2.555)	0.541 (0.217,1.347)
*p-value*	0.036*	0.473	0.615	0.207	0.508	0.187
**Housing wall material** *(cement wall vs*. *metal sheet walls)*						
*Adjusted Coefficient or Odds Ratio (95% CI)*	0.155 (-1.496,1.806)	0.205 (0.009,0.401)	-0.129 (-0.366,0.107)	1.194 (0.605,2.358)	0.552 (0.258,1.183)	2.780 (0.681,11.35)
*p-value*	0.854	0.040*	0.284	0.609	0.127	0.154
**Water source** *(piped into household vs*. *public tap/piped into yard)*						
*Adjusted Coefficient or Odds Ratio (95% CI)*	-1.933 (-2.984,-0.881)	0.127 (-0.018,0.273)	-0.171 (-0.367,0.025)	0.689 (0.435,1.089)	1.006 (0.573,1.766)	2.298 (1.078,4.899)
*p-value*	0.000**	0.086	0.087	0.111	0.984	0.031*
**Depressive symptoms (PHQ-9 score)** ^**c**^						
*Adjusted Coefficient or Odds Ratio (95% CI)*	*Outcome variable for Model 1*	-0.012 (-0.025,0.001)	0.029 (0.014,0.044)	1.023 (0.983,1.065)	1.035 (0.988,1.085)	1.066 (0.994,1.143)
*p-value*		0.063	0.000**	0.259	0.142	0.073
**log(Household capacity to pay)** ^**c**^						
*Adjusted Coefficient or Odds Ratio (95% CI)*	-0.599 (-1.212,0.013)	*Outcome variable for Model 2*	0.392 (0.275,0.510)	1.716 (1.264,2.330)	0.780 (0.570,1.069)	1.048 (0.661,1.660)
*p-value*	0.053*		0.000**	0.000**	0.123	0.843
**log(Household food expenditure)**^**c**^						
*Adjusted Coefficient or Odds Ratio (95% CI)*	0.852 (0.400,1.304)	0.237 (0.163,0.311)	*Outcome variable for Model 3*	1.113 (0.888,1.395)	0.957 (0.718,1.277)	0.755 (0.524,1.087)
*p-value*	0.000**	0.000**		0.353	0.766	0.130
**Household debt** *(debt in household vs*. *no debt)*						
*Adjusted Coefficient or Odds Ratio (95% CI)*	0.523 (-0.496,1.542)	0.290 (0.142,0.438)	0.106 (-0.093,0.304)	*Outcome variable for Model 4*	2.224 (1.238,3.996)	76.58 (27.93,210.0)
*p-value*	0.315	0.000**	0.297		0.007**	0.000**
**Reduce frequency or size of meals** *(reduction in meal size or frequency in response to financial distress vs*. *no reduction)*						
*Adjusted Coefficient or Odds Ratio (95% CI)*	0.833 (-0.461,2.127)	-0.127 (-0.290,0.036)	-0.029 (-0.251,0.192)	2.171 (1.209,3.899)	*Outcome variable for Model 5*	1.784 (0.726,4.383)
*p-value*	0.207	0.126	0.796	0.009**		0.207
**Draw up retail shop accounts** *(draw up retail shop account in in response to financial distress vs*. *no retail shop account)*						
*Adjusted Coefficient or Odds Ratio (95% CI)*	1.746 (0.032,3.460)	0.039 (-0.189,0.266)	-0.211 (-0.476,0.054)	70.21 (27.07,182.1)	1.684 (0.774,3.666)	*Outcome variable for Model 6*
*p-value*	0.046*	0.739	0.119	0.000**	0.189	
R^2^	0.164	0.223	0.158	0.179	0.098	0.295

Model 1: Multivariable predictors of depressive symptom severity (PHQ-9 score)

Model 2: Multivariable predictors of log-transformed annual capacity to pay per adult equivalent

Model 3: Multivariable predictors of log-transformed food consumption per adult equivalent

Model 4: Multivariable predictors of debt in the household

Model 5: Multivariable predictors of reducing size of frequency of meals in response to financial distress

Model 6: Multivariable predictors of drawing up shop accounts in response to financial distress

PHQ-9: Patient Health Questionnaire 9-item

^**a**^Multiple linear regression model (Models 1–3): adjusted regression coefficients and 95% CI are reported. For continuous predictor variables, the coefficient indicates the increase or decrease in the outcome variable per unit increase in the predictor; for categorical predictor variables, the coefficient indicates the difference in the outcome variable between the specified group and the comparison group indicated in brackets next to the predictor variable name.

^b^Logistic regression models (Models 4–6): adjusted odds ratios and 95% CI are reported. For continuous predictor variables, the odds ratio indicates the increased or decreased odds of the outcome variable per unit increase in the predictor; for categorical predictor variables, the odds ratio indicates the increased or decreased odds of the outcome variable between the specified group and the comparison group indicated in brackets next to the predictor variable name.

^c^Included as a continuous variable

*p<0.05

**, p<0.01

*Adjustments to co-efficients and odds ratios for log*-*transformed variables*[44]

*For linear regression*:

- Where predictor variables are log-transformed (and the outcome variable is not): the coefficient of the predictor variable was back transformed using the following equation: β(coefficient) x log(1.1) to estimate the effect on the outcome for a 10% change in the predictor
- Where both the predictor and outcome variables are log-transformed, we use the equation: (1.10) ^β(coefficient of transformed predictor)^ to estimate the effect on the outcome variable for a 10% change in the predictor variable
- Where the outcome variable is log-transformed, but the predictor variable is not: the coefficient of the predictor variable was exponentiated: exp ^β(coefficient)^ to reflect the change in the outcome variable for a one unit change in the predictor variable. If the predictor variable is dichotomous, the exponentiated coefficient is the ratio of the expected geometric mean for the one group over the expected geometric mean of the comparison group, when the other variables are held at a fixed value.

For logistic regression

- Where predictor variables are log-transformed the outcome variable is not in logistic regression, the following was applied: the OR in the output is xx, then the coefficient is log(xx). A 10% increase in the predictor variable corresponds to log(xx)* log(1.1) change in the outcome variable. The odds ratio corresponding to this change is exp(log(xx)*log(1.1))

In model 2 (relating to household capacity to pay), multivariable analysis demonstrated that married household heads (p<0.01), housing in which walls were constructed of cement (p<0.05), higher food expenditure (p<0.01) and having household debt (p<0.01) were independently associated with higher household capacity to pay. Inversely, larger household sizes (p<0.01) were independently associated with lower household capacity to pay per adult equivalent. Whilst model 2 found that worse (higher) PHQ-9 scores were associated with lower household capacity to pay per adult equivalent, this relationship was not significant at the 95% confidence level (p<0.10). Household capacity to pay was 29% higher for married household heads compared to unmarried household heads. For every one-unit increase in household size, capacity to pay per adult equivalent decreased by 6%. Household capacity to pay was 23% higher among households in which the walls were constructed of cement when compared to those constructed of metal sheets. Surprisingly, household capacity to pay increased by 34% for households with debt in comparison to those without debt.

Model 3 (on food health expenditure) demonstrated that worse (higher) PHQ-9 scores (p<0.01) and higher household capacity to pay (p<0.01) were independently associated with higher food expenditure. For a one unit increase in PHQ-9 scores, we would expect a 3% increase in food expenditure; similarly, a 10% increase in household capacity to pay would be associated with a 3% increase in food expenditure.

In model 4 (debt), multivariable analysis demonstrated that larger household sizes (p<0.05), higher household capacity to pay (p<0.01), and coping with financial distress by reducing the frequency or size of meals (p<0.01) and by drawing up retail shop accounts (p<0.01) were independently associated with households having debt. Depression symptoms were not significantly correlated with debt within the household. For a one-unit increase in household size, we would expect a 14% increased odds of having household debt. Starkly, the odds of debt are 117% higher among households who are reducing the frequency and size of meals in response to financial distress and 70 times higher for households who have drawn up retail shop accounts. A 10% increase in household capacity to pay increased the odds of debt affecting households by 5%.

Model 5 (frequency or size of meals) demonstrated that lower age of the household head (p<0.05) and debt affecting households (p<0.01) were independently associated with responding to financial distress by reducing the frequency or size of meals. For a one unit increase in household age, the odds of reducing the frequency of meals reduced by 2%, whilst for households affected by debt, the odds of reducing the frequency or size of meals in response to financial difficulty increased by 122%.

In model 6 (drawing up shop accounts), household debt affecting households (p<0.01) and having water piped directly into the household (as opposed to making use of a public water source) (p<0.05) were found to be independently associated with drawing up retail shop accounts in response to financial distress. Although higher (worse) PHQ-9 scores were not found to be independently associated with drawing up retail shop accounts in response to financial distress at the 95% confidence level, higher PHQ-9 scores appeared to increase the odds of drawing up a shop account (p<0.10). The odds of drawing up a retail shop account in response to financial distress were 77 times higher for households with debt in comparison to households with no debt; and 2.3 times higher for households with water piped directly into the household.

## Discussion

This study provides new evidence on the economic burden of depression symptoms in South Africa. We assess this burden at the level of the household; by severity of symptoms; and include households that are also suffering the impacts of chronic physical health conditions. In this way, we add to existing literature that has predominantly focused on individual-level economic costs of depression in those with a diagnosis of major depression and without comorbid conditions [9]. Consideration of the household-level impacts of depression symptoms may provide an understanding of whether financial risk protection efforts could mitigate the negative economic consequences of depression to households.

Symptoms of depression were found at higher than anticipated rates, with 94% of the screened PHC attenders found to have some degree of depression symptoms –48% of whom met the clinical threshold score of 10, indicating probable cases of major depression. This is in keeping with evidence suggesting high comorbidity of depression with hypertension, HIV and diabetes [45–47]. The PRIME cohort study—which provided the recruiting ground for this current study—found that the majority of participants were attending the PHC facility for treatment related to HIV and hypertension [48]. These findings underscore the importance of integrating mental healthcare into care for other chronic physical conditions at the primary health care level.

According to the World Bank, when deciding between monetary measures of poverty, consumption has been found to be more closely related to a person’s well-being with regards to having enough resources to meet current basic needs; with expenditure (consumption) data being more reliable than income data in household survey research [49, 50]. Consumption is also more appropriate in economies with large informal sectors, such as South Africa, as such household consumption is often used as a proxy of *effective* income [50]. In 2015, the South African national poverty line stood at USD 68.50 (ZAR922) per capita, per month; and this study has found that the entire sample of households fell below this line, using both metrics of household income and effective income (i.e. household consumption) [51].

Surprisingly, this study also found that whilst all households affected by depression symptoms had lower effective incomes (consumption), when compared to households unaffected by depression symptoms, neither household consumption nor income emerged as being significantly associated with depression symptom severity, nor was depression symptom severity significantly associated with consumption or income, through bivariate and multivariable analyses. There are several possible explanations for these findings. Firstly, given the limited variability in absolute incomes and consumption across the sample, with monthly consumption per capita varying by only USD18.6 per capita, per month, between households with the lowest median consumption (minimal depression symptoms), and those with the highest median consumption (no depression symptoms), the sample was likely too homogenous with respect to these metrics to detect significant differences. Secondly, while it is hypothesized that earnings decrease as a result of the productivity impacts of poor health, thereby resulting in an overall decrease in the resources available for consumption; total resources available to meet a household’s needs may not decrease at the same rate as that of income due to other mitigating practices such as private income transfers from friends and family or households decumulating their assets or borrowing [52]. This study found that sampled households reported high levels of debt ranging from 34%-61%, with the proportion of households reporting debt increasing with depressive symptom severity. Further, drawing up retail shop accounts in response to financial distress was found to be independently associated with worse depression symptoms. Taken together, households may be accruing debts in the short-term to maintain their overall consumption needs–however these practices are known to have detrimental long-term, intergenerational effects associated with lifelong repayment [12, 16, 17, 20]. This highlights the extreme vulnerability of all households included in this sample, but particularly those affected by depression symptoms.

A project of the World Bank’s Development Research Group[53] which investigated the socio-economic context of poor mental health in LMICs including Tonga, India, Indonesia, Mexico and Bosnia and Herzegovina also found no clear relationship between mental health and per-capita household consumption [54]. Similarly, Babiarz et al (2017) found that households did not experience a major change in consumption following a diagnosis of a severe or mild physical health condition, but if the household head was diagnosed with a psychological or mental health problem, consumption expenditure declined by 6–7% [52]. In addition Babiarz et al (2017) found that younger household heads were more likely to become unemployed or become financially dependent on other family members following a diagnosis of a mental health problem [52]. The limitations of our study design meant that we were unable to identify whether the individual affected by depression symptoms was the household head, potentially masking the relationship between depression symptoms and consumption.

An important and significant finding of this study relates to the relationship between household financial capacity to pay and depression symptom severity. Increasing financial capacity to pay was found to be independently and significantly associated with lower depression symptom severity. The association between increased financial capacity to pay and lower depression severity may be explained by two potential causal pathways: the social causation pathway (by which increased financial capacity to pay reduces depression severity for example by reducing financial stress or increasing the available resources to cope with the consequences of negative life events); or the social drift pathway (by which lower depression symptom severity increases financial capacity to pay, for example through improved work performance or increased income generating opportunities associated with improved social or economic functioning). However, the cross-sectional nature of our data does not allow us to draw clear conclusions in this regard.

A household’s financial capacity to meet its needs is arguably not a function only of its income (whether measured using income itself or consumption, as a proxy). The rationale for the use of capacity to pay as an important metric for assessing the economic circumstances of households is based on the acceptance that before a household can make decisions regarding how and where to spend its resources, its basic subsistence needs must be met [50]. In this study, consistent with others in the field, food expenditure was used as a proxy for subsistence and, noting the aforementioned reliability issues associated with income data, consumption was used as a proxy for effective income. The household’s financial capacity to meet all its non-food household needs was calculated as effective income net of food consumption. These findings therefore suggest that where households have a larger amount of resources to meet their needs, the severity of depression symptoms among the affected household member is reduced. Other factors such as receipt of social grants which would contribute to effective income were also associated with higher depression symptom severity. While this finding may seem surprising given the presumed financial protection offered through government grants, eligibility for these grants in South Africa is based on demonstration of illness and being below a particular income threshold for the Child Support Grant (which may independently predict depression). Given the outlined benefits of using consumption measures as a proxy for income, this may explain why capacity to pay has been more responsive to depression symptoms in our sampled households.

The analysis further highlights the significance of considering other variables in relation to the association between depression symptoms and poverty, with data demonstrating that higher food expenditure and drawing up retail shop accounts in response to financial distress were also both independently associated with higher depression symptom severity. Consideration of this finding in the context of Engel’s law [55] can provide a possible explanation: as income rises, the proportion of income spent on food falls, signifying improvements in the satisfaction of needs extending beyond basic needs such as food.

Further, housing conditions, such as having piped water directly into the household (as opposed to making use of public water sources) were independently associated with lower depression symptom severity, while housing in which walls were constructed of cement (rather than metal sheeting) was predictive of higher household capacity to pay, underlining the importance of addressing the social determinants of mental health, particularly by improving structural characteristics of neighborhoods and access to infrastructure [56]. Another unforeseen finding of these analyses was that households with debt have higher capacity to pay. Having debt, in this case, may serve as an indication of the households’ ability to access credit through formal employment, and therefore reflect increased financial freedom. The existence of debt however has ripple effects on household coping practices including responding to financial distress by reducing the frequency or size of meals and going further into debt by drawing up retail shop accounts. Although higher depression symptom severity was not found to be associated with drawing up retail shop accounts in response to financial distress at the 95% confidence level, worse depression symptoms did appear to increase the odds of drawing up a shop account (p<0.10).

The present findings should be considered in the context of the study’s limitations. Firstly, given that the study is cross-sectional, it is limited by recall bias and causal inferences cannot be drawn. The extent to which we can infer social causation or social drift mechanisms in the relationships we have identified is therefore limited. Secondly, the study only collected information on depression symptoms and is not a comprehensive assessment of all mental disorders; as such, it does not fully capture the impact of more severe mental disorders. A third limitation of this study was the inability to identify the role of the depression-symptom affected individual within the household, or to index illnesses affecting other household members. A fourth limitation of the study is potential endogeneity, specifically that confounding of unobserved variables in our analysis could not be controlled for. However, the study did attempt to collect a wide range of indicators relating to the socio-economic conditions of households and wealth of households. One factor that could not be controlled for is the selection bias that is likely present in the study due to the recruitment strategy of identifying patients attending primary health care facilities. These participants have higher health seeking behavior and therefore may also be different from the general population with regards to both their income and likelihood of depression given its links with delayed care seeking, and therefore the generalizability of the findings cannot be assured. It is important however to note that over 90% of participants screened positive for depression symptoms, 48% of which were diagnosed. Furthermore, participants reported very low incomes and asset-based wealth. While care is free of charge at primary health care level in South Africa, participants accessing the services may have been better placed to cover travel related costs. The study is robust however in the sense that the diagnosis for depression was made using both the application of a screening tool by a trained interviewer with agreement and diagnosis by an experienced clinician.

## Conclusion

This study is the first of its kind carried out in South Africa, identifying household economic factors associated with increased depression symptom severity on a continuum; and demonstrating that financial risk protection efforts are needed across this continuum. These findings need to be included in policy considerations to achieve effective protection from economic vulnerability for households affected by depression symptoms, particularly in light of high-levels of co-morbidities in South Africa. This can be achieved both by routinizing screening for depression and integrating treatment for mental disorders into chronic disease management; expanding eligibility for social protection mechanisms and supporting development efforts towards improving the conditions by which people live.

## Financial disclosure statement

The research leading to these results is funded by the European Union's Seventh Framework Programme (FP7/2007-2013) under grant agreement n° 305968. The funder had no role in study design, data collection and analysis, decision to publish, or preparation of the manuscript. SD is a staff member of the University of Cape Town and a PhD scholarship beneficiary funded by the South African Medical Research Council under the Bongani Mayosi National Health Scholars Programme. The authors alone are responsible for the views expressed in this publication and they do not necessarily represent the decisions, policy or views of the University of Cape Town, the South African Medical Research Council or the World Health Organization.

## Supporting information

S1 File Description and construction of household economic measures. (DOCX)

S2 File Dataset. (XLS)

S1 Table Sociodemographic characteristics of the included and excluded households. (DOCX)
