# Supplementary material for: The household economic costs associated with depression symptoms: A cross-sectional household study conducted in the North West province of South Africa
Source: PLoS One. 2019 Nov 5;14(11):e0224799. doi: 10.1371/journal.pone.0224799 (PMC6830818; doi:10.1371/journal.pone.0224799)
Supplement: S1 File — (DOCX) [file pone.0224799.s001.docx]

**S1 Supporting Information. Description and construction of household economic measures.**

*Household Income* included all reported household income received from wages, rental property, self-employment (informal trade), savings and cash transfers into the household (from: other households, community organizations or the government (e.g. social grants)). With the exception of cash transfers, which asked households to report totals received annually, all households were given the option of reporting income received daily, weekly, monthly or annually. Regarding *household* *consumption*, for items that were likely to be purchased on a frequent basis (e.g. food) households were asked how much they spent during the last week; a monthly recall period was applied for less frequent spending items like clothing, housing, frequent health items (e.g. consultation fees, medication) and transport; and an annual recall period was applied for very infrequent expenditures such as durable household assets, vehicles, education, cultural rituals and infrequent health items (hospitalizations, ambulatory costs, health devices such as eyeglasses).

After standardizing the time period to annual, total household food (subsistence) consumption was subtracted from total household income as a measure of *households’* *capacity to pay*. These financial variables were adjusted for household size and composition to ensure all comparisons generated would be based on a per adult equivalency (per capita) basis, using the OECD modified scale [25, 40]. This approach assigns a value of 1.0 to the first adult household member (or household head), a value of 0.5 to each additional adult household member and a value of 0.3 to each child household member to standardized estimates and account for the varying resource needs of adults and children in the household, and the economies of scale associated with sharing household resources [25, 40]. All household financial data were converted to United States Dollars (USD) using the 2015 average annual exchange rate (the year data collection was conducted) reported by the United States Department of Treasury for South Africa (1 USD = ZAR 13.46) [41]

A range of household assets were used to generate a *household asset score* using Multiple Correspondence Analysis (MCA). Household measures of socioeconomic status were collected to create the asset index. These included: household floor material, cooking location, access to electricity or solar energy, land or property ownership, access to improved drinking water and sanitation sources, receipt of regular income, as well as possession of a range of household assets including bicycle, car, motor-cycle, cell-phone, watch/clock, landline, valuables such as jewellery, as well as five contextualized furniture items, and nine contextualized household appliances; three each reflecting likely ownership by poor, middle-wealth and rich households. MCA as opposed to Principal Components Analysis (PCA) was used to create the asset index as MCA makes fewer assumptions about the underlying distributions of indicator variables and is more suited for the analysis of categorical variables [25, 42-44]. Wealth quintiles were generated based on these scores.

For the assessment of *household-use of distress financing strategies in response to financial difficulty*, summary variables were generated based on the household report of: withdrawing children from school, reducing healthcare use, restricting the size or frequency of meals, or drawing up accounts at retail outlets in response to financial distress over the past three years. Similarly, for the assessment of the presence of *household debt*, summary variables were generated based on the household report of debt in the household.
