# Supplementary material for: The household economic costs associated with depression symptoms: A cross-sectional household study conducted in the North West province of South Africa
Source: PLoS One. 2019 Nov 5;14(11):e0224799. doi: 10.1371/journal.pone.0224799 (PMC6830818; doi:10.1371/journal.pone.0224799)
Supplement: S1 Table — (DOCX) [file pone.0224799.s003.docx]

| **S1: Sociodemographic characteristics of the included and excluded participants** | | | |
| --- | --- | --- | --- |
| Characteristics | Included (n=534) | Excluded (n=62) | p-value |
| Head Gender  (% female) | 52 | 48 | 0.584 |
| Head Marital Status  (% married) | 27 | 27 | 0.796 |
| Head Education  (% no formal education) | 12 | 11 | 0.923 |
| Head Age (mean age) | 52 | 54 | 0.130 |
| Household size (mean number of members) | 4 | 4 | 0.183 |
| Indexed Patient Gender  (% female) | 79 | 75 | 0.490 |
| Indexed Patient Marital Status  (% married) | 21 | 19 | 0.108 |
| Indexed Patient Education  (% no formal education) | 8 | 7 | 0.944 |
| Indexed Patient Age (mean age) | 47 | 49 | 0.265 |
